# Proliferation and Differentiation Potential of Human Adipose-Derived Stem Cells Grown on Chitosan Hydrogel

**DOI:** 10.1371/journal.pone.0120803

**Published:** 2015-03-06

**Authors:** Tanya Debnath, Sutapa Ghosh, Usha Shalini Potlapuvu, Lakshmi Kona, Suguna Ratnakar Kamaraju, Suprabhat Sarkar, Sumanlatha Gaddam, Lakshmi Kiran Chelluri

**Affiliations:** 1 Transplant Immunology & Stem Cell Laboratory, Global Hospitals, Hyderabad, India; 2 Nanomaterials Laboratory, I & PC Division, Indian Institute of Chemical Technology, Hyderabad, India; 3 Department of Bariatric Surgery, Global Hospitals, Hyderabad, India; 4 Department of Genetics, Osmania University, Hyderabad, India; University of California, San Diego, UNITED STATES

## Abstract

Applied tissue engineering in regenerative medicine warrants our enhanced understanding of the biomaterials and its function. The aim of this study was to evaluate the proliferation and differentiation potential of human adipose-derived stem cells (hADSCs) grown on chitosan hydrogel. The stability of this hydrogel is pH-dependent and its swelling property is pivotal in providing a favorable matrix for cell growth. The study utilized an economical method of cross linking the chitosan with 0.5% glutaraldehyde. Following the isolation of hADSCs from omentum tissue, these cells were cultured and characterized on chitosan hydrogel. Subsequent assays that were performed included JC-1 staining for the mitochondrial integrity as a surrogate marker for viability, cell proliferation and growth kinetics by MTT assay, lineage specific differentiation under two-dimensional culture conditions. Confocal imaging, scanning electron microscopy (SEM), and flow cytometry were used to evaluate these assays. The study revealed that chitosan hydrogel promotes cell proliferation coupled with > 90% cell viability. Cytotoxicity assays demonstrated safety profile. Furthermore, glutaraldehyde cross linked chitosan showed < 5% cytotoxicity, thus serving as a scaffold and facilitating the expansion and differentiation of hADSCs across endoderm, ectoderm and mesoderm lineages. Additional functionalities can be added to this hydrogel, particularly those that regulate stem cell fate.

## Introduction

Biomaterial provides scaffold and mimic extracellular matrix (ECM). It affords great potential in regenerating large tissue injuries and focal defects [1, 2]. Hence, it is imperative to understand the local niche of the tissue injury to be able to repair them with biocompatible tissue constructs. Langer and Vacanti have paved the way to utilize these cell laden biocompatible materials in regenerating the tissues which mimic natural organs [3]. Since then, modulation of matrix properties for tissue engineering applications has been of great interest. The study therefore addresses the chitosan based hydrogel, optimized for its role in modulating the site of cellular niche [4].

Stem cell-based therapies to treat soft tissue defects due to trauma, tumor resection, aging, and congenital abnormalities significantly depend on the availability of organs. Paucity of the available organs has been the driving force to develop alternate strategies that utilize the technological advances made in the areas of tissue engineering [5, 6]. Adipose tissue source is a major source of attention which has least ethical implications and increased donation. Current research is focused on utilizing adipose tissue to overcome the limitations, and combine the functionalized biomaterial for the intended purpose. Further, adipose tissue has demonstrated trilineage differentiation potential to osteocytes, chondrocytes and adipocytes representing an ideal source for autologous cells [7, 8].Adipose tissue obtained from omentum, is comparable to bone marrow and sub cutaneous fat in terms of proliferation and differentiation potential. hADSCs make upto 7% of the cells in a lipoaspirate. Collagenase-digested adipose tissue yields roughly about 50,000 cells per ml of adipose tissue, which is 100-fold higher than that of bone marrow-derived MSC [9–11]. However, tissue repair and regeneration is a complicated process. For tissue engineering applications, biomaterials often serve as scaffold for a specific cell type. Furthermore, the biomaterial must integrate mechanically and physiologically with the repair tissue, or should be degraded without leaving gaps or fissures in the tissue that encapsulates the stem cells. Good cell viability and conserving specific cell phenotype are additional considerations regarding the construction of a three-dimensional (3D) microenvironment for stem cells to mimic in vivo conditions [12, 13].

Synthetic materials that are commonly used for tissue engineering applications include poly- lactic acid, poly-glycolic acid, or a combination of the two. These are fibrous, non-toxic, and biodegradable molecules, and can be easily manipulated without encouraging cell adhesion [14]. In the present study, chitosan hydrogel, an amino polysaccharide copolymer of 1, 4-D- glucosamines and N-acetyl glucosamines derived from chitin by alkaline or enzymatic deacetylation, was characterized. A chitosan hydrogel scaffold is hydrated and has been shown to provide a supporting matrix for human adipose-derived stem cells (hADSCs) [15].In contrast, unmodified chitosan can only be dissolved in acidic solutions due to its strong intermolecular hydrogen bonds, and this limits its applications. The crosslinking reaction is mainly influenced by the size and type of crosslinker agent and the functional groups of chitosan. The smaller the molecular size of the cross linker, the faster the cross linking reaction. This enables easy diffusion. Depending on the nature of the cross-linker interactions, a network of covalent or ionic bonds is formed [16]. Glutaraldehyde is an effective bi-functional crosslinking agent that is water soluble, highly efficient, and economical. Glutaraldehyde can be optimized to subliminal toxicity and can serve as an effective cross linker to natural polymers such as chitosan-gelatin [17]. The current study is focused on chitosan derivatives gelled via glutaraldehyde crosslinking [18–20].

Herein, we hypothesize that an alternate tissue source such as adipose tissue would provide an ideal cell source for tissue engineering applications. In the present study, a preliminary characterization of glutaraldehyde-crosslinked chitosan hydrogel is presented with an objective to evaluate its use in adipose derived stem cell culture models in vitro, and its potential differentiation capabilities of the ectoderm, mesoderm and endoderm lineages.

## Material and Method

Dulbecco's Modified Eagle Medium (Low glucose-DMEM; 4.5mmol/L glucose), type I collagenase, and fetal bovine serum (FBS) were purchased from Gibco (BRL, USA). All antibodies were purchased from BD Bioscience (San Jose, USA). Chemicals were purchased from Sigma (St. Louis, USA). Prior written informed consent was obtained from donors ranging in age from 26–57 years, undergoing liposuction procedures for morbid obesity. The study was approved by the Global Hospitals, Institution Ethical Committee (IEC), Ref no. GMERF/BS/SAC/IEC/IC_SCR2014/01. The adipose tissue for the study was collected in sterile DMEM media supplemented with antibiotics, in accordance with the code of ethics of the World Medical Association (Declaration of Helsinki).

### Preparation of hydrogel

Chitosan (0.50 g) was dissolved in 30 mL distilled and degassed 1% (wt) acetic acid solution, and then 1.50 g poly (acrylic acid) was added. When a homogeneous solution was achieved, it was placed in a 60°C water bath. Glutaraldehyde (0.5% v/v) solution was then added to the mixture and cross-linking was allowed to proceed. After 1 h, NaOH solution (1N) was added to achieve a pH of 7. Ethanol (300 mL) was subsequently added to the gelled product with stirring. After complete dehydration of the hydrogel for 24 h, the hardened semi-IPN hydrogel product was filtered, washed with fresh methanol, and dried at 50°C.

### Microstructure observations

A cross section of the chitosan-based hydrogel was observed using scanning electron microscopy (SEM). For these studies, the samples were fixed in 2.5% glutaraldehyde in 0.05 M phosphate buffer (pH 7.2) for 24 h at 4°C, and then were post-fixed in 2% aqueous osmium tetraoxide in the same buffer. After 2 h, the samples were dehydrated in a series of graded alcohols and were dried to a critical point using an electron microscopy science CPD unit. These samples were then mounted over the tubes with double-sided conductivity tape, and a thin layer of gold metal was applied for ~3 min over each sample using an automated sputter coater (JEOL JFC-1600). Samples were then scanned at various magnifications using a scanning electron microscope (Model: SEM Hitachi- S520, Japan; Oxford Link ISIS-300 UK) of the Electron Microscopy Center, IICT (Hyderabad, India).

### Swelling behavior of the hydrogel

The swelling ratios and swelling kinetics of the hydrogel samples were measured gravimetrically. Briefly, the weights of swollen hydrogels were measured after the excess water was wiped from their surfaces with moistened filter paper. Three measurements from three parallel specimens from the same hydrogel were then obtained for each sample, and an average weight was determined. Swelling ratios were measured by immersing hydrogel samples in water baths at different temperatures for different time intervals. The weight of each swollen sample was recorded at each immersion temperature with various immersion time intervals.

The swelling ratio, S, was calculated as follows:

$$S=\frac{W_{t}-W_{d}}{W_{d}}$$

To obtain the equilibrium swelling ratio, Seq:

$$S_{eq}=\frac{W_{e}-W_{d}}{W_{d}}$$

Where, Wt and We are the weights of the swollen hydrogels at a time interval, t, or at an equilibrium state under a given condition, respectively; Wd is the dry weight of the hydrogel.

### Isolation and characterization of adipose-derived stem cells

Omentum tissue was obtained from donors ranging in age from 26–57 years who were undergoing liposuction procedures for morbid obesity and were pooled to overcome the age variability and impact on the proliferation and differentiation potential. Discarded fat tissue was immediately collected in sterile DMEM media supplemented with antibiotics. Tissue fragments were then washed vigorously with PBS, and were digested with type I collagenase (1 mg/mL) for 30–60 min in a humidified atmosphere of 95% air and 5% CO_2_ at 37°C with gentle agitation. The digested tissue was then filtered through a 40 μm cell strainer and the cells obtained were centrifuged at 1,800 rpm for 5 min. The resulting cell pellet was resuspended in L-DMEM supplemented with 10% FBS, penicillin (100 IU/mL), streptomycin (100 IU/mL), gentamycin (50 IU/mL), amphotericin B (2.5 μg/mL), and basic fibroblast growth factor (bFGF) (10 ng/mL). Cells were then plated at a density of 1 x 10^6^/cm^2^ in polystyrene T25 culture flasks and were incubated in a humid 5% CO_2_ incubator at 37°C. After 48 h, non-adherent cells were discarded and the adherent cells were replenished with complete medium every 3 d. The cells were cultured for 10 d. Adipose tissue-derived stem cells were harvested at 80% confluence and were seeded at a concentration of 1 x 10^3^ cells/mL on petri dishes. At various time points (e.g., 24 h, 48 h, 72 h, 96 h, 120 h, and 144 h) growth kinetics was assayed (n = 5).

### hADSCs profiling by flow cytometry and immunocytochemistry

Cell surface antigen expression by hADSCs (n = 6) at each passage was detected using flow cytometry. Epitopes such as: CD90 (FITC), CD34 (PE), CD73 (APC), CD45 (FITC), and HLA-DR (PE) were evaluated for positive versus negative expression using Cell Quest software (Becton Dickinson). Adipose derived stem cells were further characterized by immunocytochemistry to detect the presence or absence of CD90, CD73 and HLA-DR, CD45, and CD34 markers, respectively (n = 3). Briefly, MSCs (2x10^3^cells/mL) were grown on cover slips in a humidified atmosphere. After, 18 h the cover slips were fixed with 3.7% paraformaldehyde for 20 min at room temperature. They were further treated with 0.1% Triton-X100 for cell permeabilization and were blocked with 5% BSA. After 1 h, the cover slips were incubated with primary antibodies against the surface markers: CD90 (1:100), CD73 (1:100), HLA-DR (1:50), CD34 (1:100), and CD45 (1:100). After incubation at 4°C overnight, the cover slips were subsequently incubated with Alexa 488- labeled secondary antibodies at room temperature. After 1 h, the cover slips were mounted on slides with mounting medium (Vectashield) containing DAPI and imaging was performed using a confocal microscope (Leica CLSM). The tri-lineage potential of MSCs was established as described previously [21].

### Growth, viability and survival rate of hADSCs on hydrogel by SEM analysis

Cellular morphology is an important phenomenon that is correlated with important biochemical functions involved in new tissue formation such as proliferation and migration of cells. The cell morphology of hADSCs on the hydrogels was therefore evaluated after encapsulation. hADSCs were seeded on a chitosan hydrogel at a final concentration of 0.1x10^6^ cells/mL. The media was changed every second day. Hydrogels were gently washed with PBS and the cells grown on each hydrogel were fixed with 2.5% v/v glutaraldehyde in 0.1 M PBS for 1 h. The hydrogels were then dehydrated in an ethanol series and were rinsed with hexamethyldisilazane. Imaging was conducted using a scanning electron microscope (Model: JOEL-JSM 5600, JAPAN) of the Department of Veterinary Pathology, Ruska Laboratories, (Hyderabad, India) at the desired magnifications to evaluate the porosity and architecture of each gel. The population doublings of the isolated hADSCs and hADSCs cultured on the chitosan hydrogel was also analyzed for significance (n = 3).

Potential viability and morphological evaluations of hADSCs(n = 3) cultured on hydrogels were assessed based on mitochondrial transmembrane integrity which is an important parameter of living cells using a lipophilic cationic probe, 5,5',6,6'-tetrachloro-1,1',3,3'-tetraethylbenzimidazolcarbocyanine iodide (JC-1).The loss of transmembrane potential is considered as a major cause of programmed cell death.

### Apoptotic/cytotoxic evaluation of hADSCs grown on hydrogel

Lactate dehydrogenase (LDH) is a soluble cytosolic oxidoreductase enzyme that is released by cells when the plasma membrane is disrupted. Furthermore, the amount of LDH that is released is proportional to the number of lysed cells. Therefore, supernatants were collected from each chamber and LDH levels were measured at 340 nm using an Olympus AU400 Automated Analyzer according to the manufacturer’s recommendation. As a positive control, cells were treated with 20μM H_2_O_2_ and then were assayed at 340 nm (n = 4).

Viability of hADSCs grown on hydrogel were assessed using MTT (3, 4, 5- dimethylthiazol-2-yl)-2–5-diphenyltetrazolium bromide) assay according to the protocol of Mossman et al. [22]. Cells that were grown in normal growth media without the gel served as a control for the interpretation of the data. All of the samples were plated in triplicate (n = 3).

Cytotoxicity associated with hydrogel was estimated using Annexin V FITC staining and the experiment was repeated thrice. As a positive control, apoptosis was induced in cells grown on the chitosan matrix with a 10 min treatment with 20μM H_2_O_2_. Images were then captured using a confocal microscope (Leica, CLSM).

### Differentiation potential of hADSCs on the chitosan hydrogel

Chondrogenic differentiation of hADSCs (n = 5) on the chitosan hydrogel was induced using high glucose DMEM supplemented with 10% FBS, 50 μg/mL ascorbic acid, 1% ITS-premix, and 0.1μM dexamethasone in the presence of 10 ng/mL TGF-β. Cells were maintained at 37°C and fresh medium was provided every third day for 21 days. The resulting pellets were fixed in 4% paraformaldehyde for 15 min, and the sections were stained with Alcian blue to visualize the acid mucopolysaccharides.

### Adipogenesis

Cells were cultured as a monolayer for 21 d on the chitosan scaffold in the presence of high glucose-DMEM, 10% FBS, and adipogenic supplements (1μM dexamethasone, 1μg/mL insulin, and 0.5mM 3-isobutyl-1-methylxanthine). To visualize adipocytes, cultures were fixed in 4% paraformaldehyde for 15 min, and were washed, stained with Oil Red O (3 mg/mL in H_2_O) for 10 min.(n = 5)

### Osteogenesis

Human ADSCs (n = 5) cultured on the hydrogel scaffold at a seeding density of 200 cells/mm^2^ were induced with osteogenic induction media containing 100nM dexamethasone, 10mMβ-glycerophosphate, and 0.1 mg/mL ascorbate-2-phosphate in the presence of 10ng/mL BMP-2. The cultures received fresh osteogenic medium every 3 d for up to 3 weeks. After three weeks, the cultures were fixed in 4% paraformaldehyde and were processed for Von Kossa staining.

### Statistical analysis

SSPS package was used for all the statistical analysis. All quantitative data are presented as mean ± standard deviation (SD). A statistical difference is considered significant at p < 0.05 using Student’s‘t’ test.

## Results

### Characterization by infrared spectroscopy and SEM

**Fig 1:**
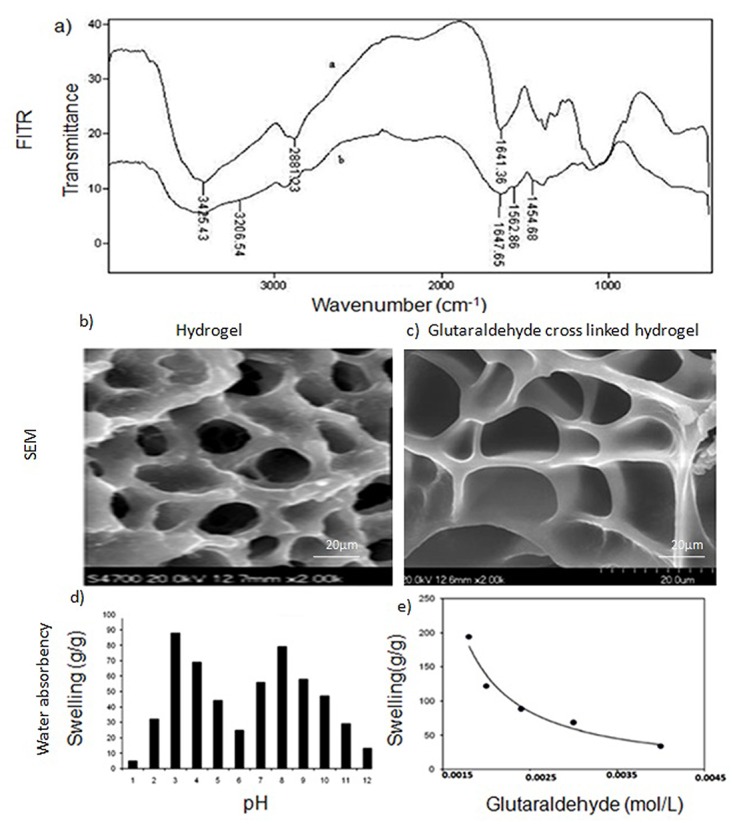
Physico-chemical properties of chitosan hydrogel. a) FTIR spectra of chitosan-based semi-IPN hydrogel. The broad band at 3425 cm^-1^ on the upper spectra corresponds to the associated-OH stretching vibrations of the hydroxyl groups. The peak at 1641 cm^-1^ corresponds to N-H deformation bending of chitosan. In the lower spectrum of the hydrogel, new peaks appeared at 3206, 1647, and 1562 cm^-1^ and these may be due to amide NH stretching, asymmetric, and symmetric amide NH bending, respectively. b&c) SEM images of the hydrogel and the glutaraldehyde cross linked chitosan-based hydrogel, respectively. Pore size of 110μm and loose architecture was observed for the latter. d) Effect of pH and cross linker concentration on water absorbency of the semi-IPN. The two sharp swelling capacity changes can be attributed to high repulsion of the-NH3+groups in acidic media and the-COO^-^groups in the basic media. However, at very acidic conditions (pH < 2), a screening effect of the counter ions (i.e., Cl^-^) shields the charge of the ammonium cations and prevents efficient repulsion. As a result, a remarkable decrease in equilibrium swelling was observed (gel collapsing). Around pH 5, the carboxylic acid component contributes as well. e) As the concentration of glutaraldelyde increases, the water absorbency of the super absorbent composite decreased. This is due to the decrease in space between the copolymer chains as the cross linker concentration increased.

The chemical structure of inter penetrating network 3 (IPN3) was investigated using Fourier transform infrared spectroscopy (FTIR). Briefly, a pellet of IPN3 was prepared by adding dried hydrogel powder to solid potassium bromide (KBr) and grinding and pressing the mixture into discs. An analysis was then performed using a Nicolet 170SX FTIR spectrometer (Madison, WI, USA) in the range of 4,000–500 cm-1 (Fig. 1a). SEM micrographs illustrated that the surface morphology of hydrogel sample was interconnected and rough. Whereas, chitosan based hydrogel with glutaraldehyde cross linking confirmed pores of 110μm in size with a smooth surface. (Fig. 1b,c).

### Effect of cross linker concentration on swelling

In hydrogels, the pores forms capillaries like channels, which are prone to adsorb solvent molecule. Depending on the pH, the functional groups present in the hydrogels may protonate or deprotonate, which causes difference in the amount of water uptake leading to its morphological change. Also due to change in pH the hydrogel may degrade or solubilise in the swelling medium.

Equilibrium swelling for the synthesized hydrogels was measured in different buffer solutions that ranged in pH from 1.0 to 12.0. The pKa of a weak polyacid is about 6.4, and ionization that occurs above this value may favour enhanced absorbency. However, when the pH is less than 6.4, particularly between pH 4 and pH 6, the majority of base and acid groups are in a non-ionized form, and hydrogen bonding between amines and carboxylic acids (as well as carboxamide groups) may lead to a form of cross-linking that is followed by a decrease in swelling (Fig. 1d).At higher pHs, the carboxylic acid groups become ionized and the electrostatic repulsive force between the charged sites (COO^-^) leads to increased swelling. Screening effect of the counter ions (Na+) can develop, and this limits the amount of swelling that occurs between pH 8 and pH 12.

Swelling ratio as a function of glutaraldehyde concentration was further investigated. Cross-linking was found to be necessary to form a superabsorbent bio-polymer, thus preventing dissolution of the hydrophilic polymer chains in an aqueous environment. Furthermore, as the concentration of glutaraldehyde increased, the water absorbency of the superabsorbent composite decreased (Fig. 1e).

### Isolation and characterization of mesenchymal stem cells from adipose tissue

**Fig 2:**
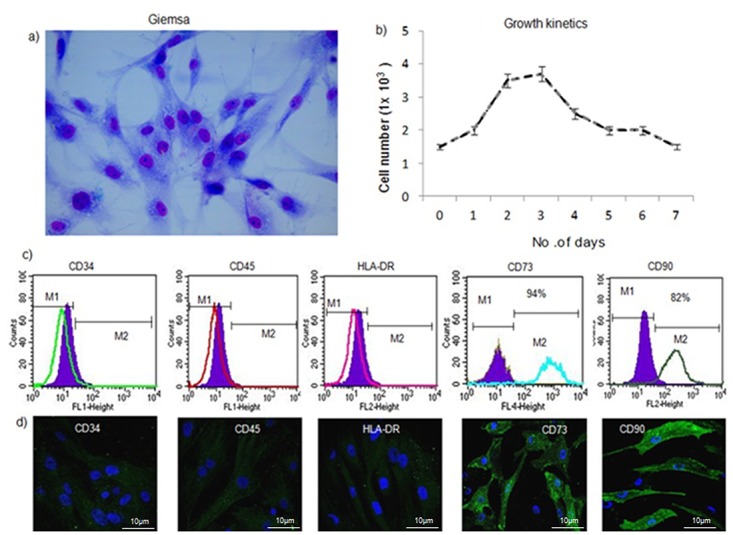
Characterization hADSCs cultures. a) Giemsa staining shows the spindle-shaped morphology of ADSCs. b) Growth curve obtained for the hADSCs (n = 5) shows an exponential growth phase. The cell number increased to 4x10^3^cells/mL after 3 days. The decline phase started after 4 days and cell number was decreased to 1.3x10^3^ cells/mL. c) Characterization of the following cell surface markers for hADSCs (n = 6) by flow cytometry: CD34-PE, CD45-FITC, HLADR-PE, CD90-FITC, and CD73-APC. The filled in histograms represent unstained negative control cells. CD34/45 markers were not expressed (0.2% and 0.3%, respectively), while low levels of HLADR expression (1.2%) were observed. hADSCs were positive for expression of CD90 (82%) and CD73 (94%), indicating a stem cell immunophenotype. d) Immunocytochemistry of hADSCs (n = 3): CD34/45and HLADR expression was negative, while CD90/CD73 expression was observed on the cell surface (shown in green).

An average yield of 1x10^6^cells/mL was obtained from 3–5 g adipose tissue. Morphologically, the hADSCs exhibited spindle shape and a fibroblast-like appearance after 14 days of culturing, as shown by Giemsa staining (Fig. 2a). The growth curve for these hADSCs (n = 5) included a lag phase but cell number increased to 3x10^3^cells/mLafter 3 days. The cells were at stationary phase for 24 h and later the cell number decreased indicating their death phase, (Fig. 2b) representing a sigmoid growth curve of hADSCs (S1 File). These hADSCs (n = 6) were also negative for CD34/45(1%), HLA-DR (3%), positive for CD90 (82%) and CD73 (94%) (Fig. 2c). A homogenous hADSCs population that resembled an MSC origin was also observed during passage #3, and these MSCs were positive for CD90 and CD73, and were negative for CD45, CD34, and HLA DR, by immunocytochemistry (n = 3) (Fig. 2d).

### Survival rate, distribution and bio-compatibilityof cells on the hydrogel

**Fig 3:**
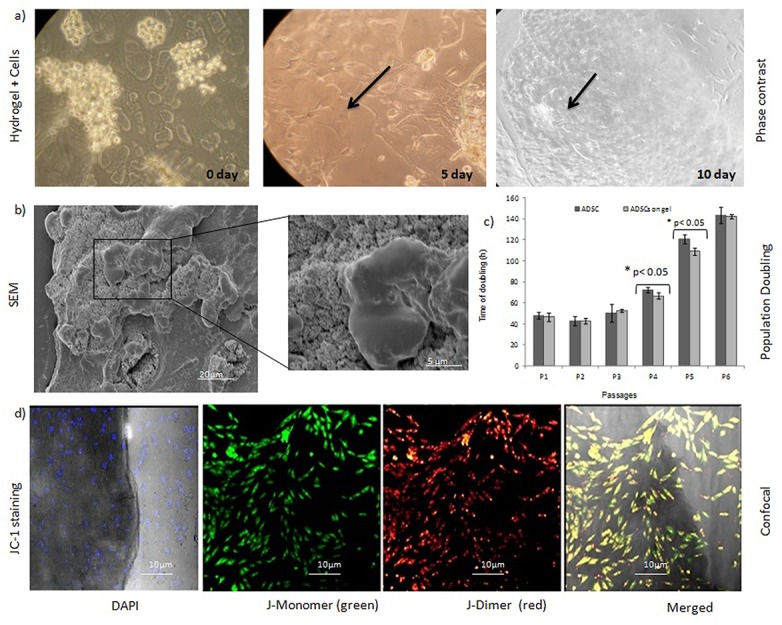
Proliferation potential of hADSCs on chitosan hydrogel. a) hADSCs cultured on chitosan hydrogel on different days. Magnification 200X. b) SEM imaging revealed uniform distribution of hADSCs on the hydrogel. Further, the cells were growing in close association with the hydrogel. c) The mean ± SD values for PDT were low when cells were grown on a hydrogel matrix. There was a significant difference in growth rates of the hADSCs on the hydrogel matrix at P <0.05 (n = 3). d) JC-1 staining showed JC-1 dimer formation within the stem cell network, thereby indicating the presence of metabolically active live cells on the hydrogel using CLSM. The JC-1 staining of cells differentiate dead from viable intact cells by their mitochondrial membrane integrity. The negative charge established by the intact mitochondrial membrane potential allows the lipophilic dye, to enter the mitochondrial matrix where it accumulates. When the critical concentration is exceeded, J-aggregates form, which appear as fluorescent orange. In non-viable cells, the mitochondrial membrane potential collapses, and the JC-1 cannot accumulate within the mitochondria. The bright field image shows actively growing stem cells present in the hydrogel (n = 3).

hADSCs grown on 10μm hydrogel sections were found to be stable throughout the entire culture period, and the cells did not undergo any contractions. It was noted the hADSCs were spindle-like shape on the hydrogel on 10th day (Fig. 3a). In SEM images, adipose-derived MSCs were found to form continuous sheets of cells and to fill the pores of the hydrogel. In addition, small, round-shaped cells were found to be embedded within the pores of the chitosan hydrogel (Fig. 3b). The average population doubling time (PDT) of the hADSCs grown on the hydrogel was 47.8 ± 0.32 h till passage 2, similar to the doubling time of hADSCs without hydrogel (n = 3). hADSCs exhibited the lowest PDT at P2 with 42.74 h, although this increased to 50.56 h at P3, and to 54 h at P4–P6 whereas, the PDT was increased to 90 h at P4 and more than 120 h at P5–P6 for isolated hADSCs without the matrix. (Fig. 3c and S2 File). Using JC-1 staining, mitochondrial integrity and compatibility of the hADSCs (n = 3) was found to be 98%. Furthermore, hADSCs retained their spindle shape morphology and exhibited > 80% viability on the hydrogel matrix compared with their growth in a monolayer cell culture. hADSCs were also observed to form a mesh on the hydrogel matrix as evidenced by the detection of intact mitochondria in fluorescent staining assays (Fig. 3d).

### Evaluation of cellular toxicity of hydrogel on hADSCs

**Fig 4:**
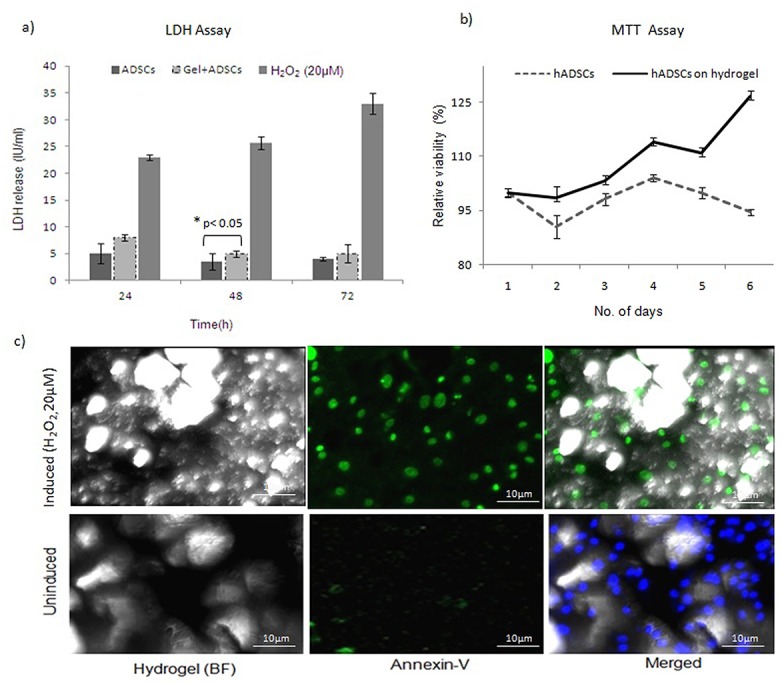
Bio-compatibility profiling of hADSCs on chitosan hydrogel. a) The results presented are the mean ± SD LDH levels detected in supernatants at different time intervals. LDH release was not detected for stem cells cultured on the hydrogel (n = 4, P <0.05). b) MTT assay was used to represent cell metabolic activity and viability of hADSCs encapsulated in chitosan matrix for 7 days (mean ± SD, n = 3). c) Apoptosis assays demonstrated that the cells did not undergo apoptosis when cultured on hydrogel. In contrast, cells induced with H_2_O_2_ underwent apoptosis (n = 3).

Cell culture supernatants were collected after 24 h, 48 h, and 72 h and were analysed for levels of LDH (Olympus AU400 Automated Analyzer). As shown in Fig. 4a, there was no significant difference in LDH assay among the hADSCs and hADSCs with matrix group after 48 and 72 h (n = 4, p<0.05). LDH release was <10 IU/mL for the tested chitosan hydrogel matrix with the cells at different time intervals indicating that the hydrogel has no cytotoxic effect on hADSCs (S3 File). The metabolic activity of mesenchymal stem cells grown on the hydrogel matrix was confirmed using MTT assay over a period of 7 d. (n = 3). The cell seeding density of 3x10^3^cells/well provided sufficient cell-cell contact. The relative cell viability of hADSCs on the hydrogel increased > 100% after 4 days (S4 File). Annexin V-FITC assays were performed to assess cytotoxicity. After hADSCs (n = 3) were cultured on the hydrogel for 7 d, no signs of phosphatidyl serine translocation were detected compared with cells induced with 20μM H_2_O_2_. These results indicate that the hydrogel tested does not induce a toxic effect on the hADSCs that were cultured with it (Fig. 4b,c).

Taken together, these results demonstrate that the hydrogel evaluated was associated with a viability of >90% and < 5% cytotoxicity. Thus, the chitosan hydrogel represents a better scaffold for cell growth.

### Differentiation potential of hADSCs

**Fig 5:**
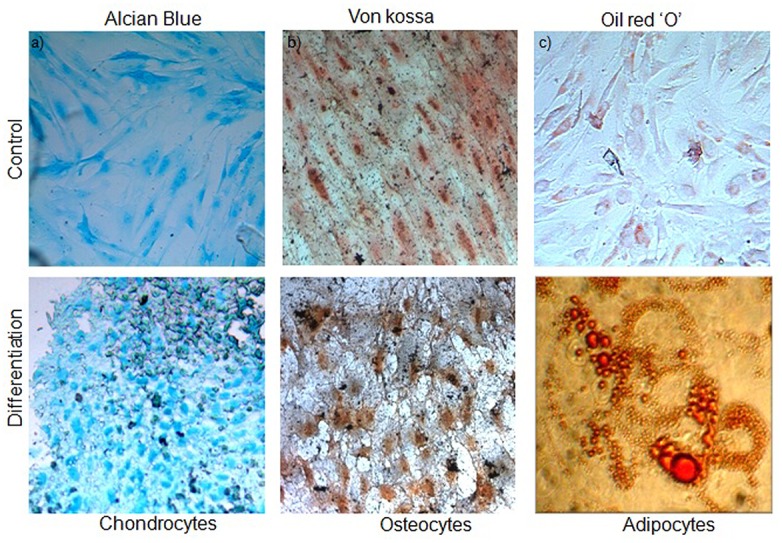
Differentiation potential of hADSCs cultured on a chitosan-based matrix. a) Alcian blue staining of acid mucopolysaccharide aggregates present in the differentiatedchondrocyte cultures grown on chitosan hydrogel. b) Calcium deposits in the ECM were stained using Von kossa staining of the differentiated osteocytes on the hydrogel matrix.c) Lipid droplets were formed on cells laden on matrix after adipogenic induction and werestained with oil red O staining. Negative staining of un-induced cultures for the respectivelineages is also shown. Magnification, 400X (n = 5).

Differentiation of fibroblastic cells obtained from adipose tissue could be directed towards adipogenic, chondrogenic and osteogenic lineages using appropriate differentiation media and supplements. For these studies, un-induced fibroblasts served as negative controls. For the chondrogenic cultures, spherical micro-mass cultures were observed, and deposits of acid mucopolysaccharides were confirmed with Alcian blue staining as shown in Fig. 5a.The cells in each group were separated by extensive regions of a diffuse extracellular matrix that had high collagen content. Similarly, under osteogenic induction conditions, a dark ECM material was detected after the induction period. Deposition of a calcified matrix was confirmed with Von Kossa staining (Fig. 5b). Adherent hADSCs which underwent adipogenic differentiation were characterized by an accumulation of cytoplasmic triglycerides that were represented as lipid droplets by Oil Red O staining (Fig. 5c). The trilineage differentiation potential of the hADSCs was confirmed in five samplesfor reproducibility.

## Discussion

Successful tissue engineering requires three key factors: (a) a unique niche with a defined medium, (b) a biocompatible scaffold with favorable structural features for cell adherence, and (c) the ability to aid in cell metabolic activities [23]. The FITR results of this study is consistent with many previous studies [24], which have demonstrated that there was a difference in FITR profile between chitosan and chitosan cross linked with glutaraldehyde. Manynew peaks appeared at 3206, 1647, and 1562 cm^–1^ and these may be due to amide NH stretchingafter cross linking. However, Oyrton et al.[25] revealed that the increase of glutaraldehyde in chitosan causes an increase of ethylene bond frequency at 1562 cm^−1^. The SEM results indicated a difference in the surface morphology between chitosan and cross linked hydrogels. SEM images in the present study revealed smooth and porous morphology of glutaraldehyde cross linked hydrogel, which is consistent with the result of previous studies [26]. Covalently crosslinked hydrogels present the crosslinking degree as the main parameter influencing important properties such as mechanical strength, swelling and drug release. Such gels generally exhibit pH-sensitive swelling and drug release by diffusion through their porous structure [27]. Therefore, hydrogels based on covalently and ionically cross linked chitosan can be considered as good candidates for the cell delivery.The structural organization of chitosan also serves as a matrix for retaining the cells at a specific site and initiating appropriate cell-to-cell interactions [28].

A chitosan-based hydrogel was created and characterized to explore various strategies for its effective use. Chitosan acts a substrate for cell attachment, proliferation by mimicking the glycosaminoglycan of the extracellular matrix [29].In particular, the use of different cross-linkers can obtain distinct architectures. Variable pH levels were also evaluated in order to obtain a stable cell/gel formulation. The goal was to provide hydration of the scaffold, yet provide a favorable niche for cells to easily adhere, survive, and proliferate into the desired cell lineage based on the growth factors provided.

Human adipose-derived MSCs share an identical phenotype with bone marrow-derived MSCs and represent a feasible, minimally invasive, and alternate tissue source for exploring the potential lineage specific differentiation of these cells into cartilage or bone. In the present study, hADSCs exhibited a similar phenotype to bone marrow cells and were also positive for MSC markers (i.e., CD90, CD73), which is consistent with previously published results. For example, in a direct comparison by Zuk et al., adipose- and marrow-derived MSCs were found to share more than 90% similarity in cell surface expression markers [30, 31].

Traditional chondrogenic inducers such as TGF-β, insulin, and dexamethasone have been reported to support chondrogenic differentiation. In contrast, isobutyl xanthine and beta glycerophosphate in the presence of BMP-2 have been found to promote adipogenic and osteogenic differentiation, respectively [32, 33]. In the present study, hADSCs were able to differentiate into specific cell lineages while being grown on a hydrogel matrix. Similarly, a recent study demonstrated that a hydrogel could induce mesenchymal cells to adopt a long striated, or spindle-shaped, morphology [34]. Formation of an adherent mesh of cells on a hydrogel also substantiates the suitability of this matrix to accommodate hADSCs as demonstrated by Song et al. [35].

However, hADSCs undergo replicate senescence as indicated by the altered morphology that was observed for passage 6 cells and beyond, when they were maintained on the hydrogel matrix. Initial cell seeding density was found to be an important parameter for controlling proliferation within the matrix under normal metabolic conditions and growth kinetics as compared with monolayer cell culturing. PDT of 42.7 h also defines the growth rate of the hADSCs with the matrix. Furthermore, our data is consistent with the population doubling values reported for stem cells derived from human adipose aspirates and marrow-derived stem cells [36]. In vitro proliferation strategies and new approaches are of great interest in regenerative medicine due to the need for a large number of cells to repair tissue-wide defects [37].

A hydrogel matrix has been shown to successfully support cell viability as well as nutrient and protein transport [38]. Data from the LDH assays performed in the present study also supports the non-toxic nature of the hydrogel matrix. Spectrophotometric-based MTT assays provided evidence that the hydrogels were able to maintain cell viability.>90%metabolically active cells were found on the chitosan hydrogel matrices than in the absence of a matrix. Annexin V FITC staining further demonstrated that cells were viable throughout the entire thickness of the matrix. Therefore, the growth and metabolic activity of hADSCs appears to be influenced by the cross-linking of chitosan, which provides appropriate and favorable cell-cell contacts within the gel and facilitates the efficient transport of oxygen and nutrients.Our results are consistent with Cheburu et al. concluding that chitosan hydrogels have no inhibitory effect on cell growth [39]. The latter may be attributed to the microsphere surfaces that exist in a hydrogel, based on the results of a recent study that demonstrated micro-cavities on scaffold surfaces improvingthe cell adhesion and eliciting differential cellular responses compared with smooth surfaces [40, 41]. Compared with other studies [42, 43] the glutaraldehyde cross linked chitosan hydrogel evaluated in the present study was found to provide a good substrate for cell attachment and controlled proliferation. Furthermore, over time, 90% of the hADSCs have differentiated into chondrocytes and osteocytes, as evidenced by the presence of acid mucopolysaccharides and calcium deposits, respectively. The finding suggests that the crosslinking affects the cell-matrix interactions and plays a critical role in supporting differentiation. Thus, this present study demonstrates chitosan hydrogel as a scaffold for hADSCs, which is biocompatible and has the propensity to incorporate the chemical cues for differentiation to three distinct lineages.It has further exhibited an innate hydrated structure. Due to these improved functionalities, it can be further tested as a macro-carrier for articular chondrocytes in the treatment of large cartilage defects.

## Supporting Information

S1 File Growth kinetics of hADSCs. Growth curve of cultured hADSCs over a period of 7 days (n = 5). The standard error bars represent mean ± standard deviation.
(XLSX)

S2 File Population doubling studies of hADSCs. Doubling time of hADSCs grown on the hydrogel matrix is plotted along with those cultured without matrix. (n = 3) The population doubling is shown on Y-axis and the number of passages is indicated on the X-axis. Graph bars represent mean ± standard deviation calculated using Student’s ‘t’ test.
(XLS)

S3 File Lactose dehydrogenase release of hADSCs cultured on chitosan hydrogel. Culture supernatants were collected from after 24 h, 48 h, and 72 h to check the release of LDH (n = 4). The dark color bar represents hADSCs cultured without matrix and the dotted bar indicates levels of LDH released when the cells were cultured on the matrix. H_2_O_2_ induced cells served as positive control. P< 0.05 represents significant changes in LDH values between induced and un-induced cultures.
(XLSX)

S4 File MTT assay. The relative viability of the cells were assessed by MTT assay (n = 3). The solid line represents growth of hADSCs on the hydrogel over period of 6 days. The Y-axis represents percentage relative viability of cells as against control. The error bars represent mean ± standard deviation.
(XLSX)
